# Epithelial/Mesenchymal Characteristics and PD-L1 Co-Expression in CTCs of Metastatic Breast Cancer Patients Treated with Eribulin: Correlation with Clinical Outcome

**DOI:** 10.3390/cancers12123735

**Published:** 2020-12-11

**Authors:** Hara Polioudaki, Anastasia Mala, Eleni Gkimprixi, Maria A. Papadaki, Amanda Chantziou, Maria Tzardi, Dimitris Mavroudis, Sofia Agelaki, Panayiotis A. Theodoropoulos

**Affiliations:** 1 Laboratory of Biochemistry, School of Medicine, University of Crete, 71003 Heraklion, Crete, Greece; polioudaki@uoc.gr (H.P.); bio2578@edu.biology.uoc.gr (E.G.); amanda_devet@hotmail.com (A.C.); 2 Department of Medical Oncology, University General Hospital of Heraklion, 71110 Heraklion, Crete, Greece; malaanastasia1@gmail.com (A.M.); mavrudis@med.uoc.gr (D.M.); agelakisofia@gmail.com (S.A.); 3 Laboratory of Translational Oncology, School of Medicine, University of Crete, 71003 Heraklion, Crete, Greece; papadaki_maria1@yahoo.gr; 4 Department of Pathology, University General Hospital of Heraklion, 71110 Heraklion, Crete, Greece; tzardi@uoc.gr

**Keywords:** metastatic breast cancer, CTCs, eribulin, PD-L1, EMT status, MET, immunocytochemistry combined to immunofluorescence, liquid biopsy, biomarkers

## Abstract

**Simple Summary:**

Circulating tumor cells (CTCs) detected in cancer patients as single cells or in clusters are of prognostic value. There is currently no information concerning the co-expression of mesenchymal markers (important for metastasis) and PD-L1 (immune system suppressor) in CTCs of metastatic breast cancer patients. In this study, we aimed to evaluate the co-expression of these markers in single and cluster CTCs and to determine if there is any relationship with patients’ outcome after eribulin treatment. CTCs were detected and phenotypically analyzed by quantifying keratins (epithelial marker), vimentin (mesenchymal marker) and PD-L1 using immunofluorescence and confocal microscopy. We found, for the first time, distinct CTC subpopulations, such as cluster and single PD-L1^+^ mesenchymal CTCs, at baseline and eight days after eribulin administration, respectively, that were associated with worse prognosis. Our study supports the role of CTC analysis in the investigation of mechanisms of resistance and disease progression in real time.

**Abstract:**

We aimed to evaluate the co-expression of PD-L1 and epithelial-mesenchymal markers in CTCs from metastatic breast cancer (MBC) patients and to determine if there is any relationship with patients’ outcome after eribulin treatment. Using cytospin preparations of peripheral blood mononuclear cells (PBMCs) from MBC patients treated with eribulin and a combination of immunocytochemistry and immunofluorescence, we quantified PD-L1, keratins and vimentin in single and cluster CTCs on days 1 and 8 of the first-treatment cycle. CTCs (*n* = 173) were found in 31 out of 38 patients. At baseline, the presence of cluster CTCs (*p* = 0.048), cluster mesenchymal CTCs (mCTCs) (*p* = 0.0003) or cluster PD-L1^+^mCTCs (*p* = 0.006) was associated with shorter overall survival (OS). In multivariate cox regression analysis, the detection of cluster mCTCs was the only parameter associated with increased risk of death (*p* = 0.024). On day 8 post-eribulin administration, PD-L1^+^mCTCs and especially single PD-L1^+^mCTCs decreased in 75% and 89% of patients, respectively. The detection of single PD-L1^+^mCTCs after eribulin treatment was correlated with shorter PFS (*p* = 0.047) and OS (*p* = 0.020). In conclusion, our study identified for the first time that cluster and single PD-L1^+^mCTCs subpopulations are of clinical significance in patients with MBC and highlighted the importance of CTC phenotyping during treatment with eribulin.

## 1. Introduction

The identification of circulating tumor cells (CTCs) in the peripheral blood is an established prognostic indicator in metastatic breast cancer [1,2,3]. CTCs are found either as single cells or in clusters, while their phenotypic analysis has shown significant intra- and inter-patient heterogeneity [4]. The detection of CTCs in clusters and their contribution in metastasis and poor outcome of patients has been highlighted in various cancers [5,6,7,8]. The molecular characteristics and properties of clusters are different from those of single CTCs [6,7] and their size considerably varies, from 2 to 100 CTCs [4,6,9].

Epithelial to mesenchymal transition (EMT) is a dynamic process that generates invasive cells and it has been suggested that CTCs undergo EMT in order to migrate to distant organs [4,10,11]. During this process epithelial cells acquire a more mesenchymal phenotype by down-regulating epithelial and up-regulating mesenchymal markers. Using quantifiable assays for epithelial and mesenchymal markers, CTCs have been categorized according to EMT status, either as exclusively epithelial, intermediate or mesenchymal [4,12]. In metastatic breast cancer (MBC), mesenchymal CTCs (mCTCs) have been detected either as single cells or multicellular clusters [4], whereas in metastatic NSCLC, mesenchymal markers were expressed in the majority of CTCs in clusters [8,9,13]. EMT has been associated with stemness properties [14,15], and interestingly, a chemoresistant CTC subpopulation co-expressing stemness and partial-EMT characteristics emerged as an independent prognostic marker in patients with MBC undergoing first-line chemotherapy [16].

Programmed cell death ligand 1 (PD-L1) is a plasma membrane receptor that is expressed by leukocytes and cancer cells [17]. PD-L1 can be induced in response to different cytokines or can be constitutively active through various oncogenic signaling pathways in cancer cells [18,19]. Binding of PD-L1 to its receptor PD-1 inhibits T cell activation. PD-L1 expression levels on cancer and/or immune cells have been associated with clinical responses to anti-PD-L1/PD-1 therapy [20]. The expression of PD-L1 in CTCs, isolated using different approaches, has been extensively investigated and reported in lung cancer [21,22]. PD-L1 was also detected in CTCs from patients with melanoma, breast, prostate and gastrointestinal cancer [23,24,25,26,27]. 

A bidirectional regulation between EMT and PD-L1 signaling has been reported in tumors from different types of cancer, including breast cancer [28,29]. In animal models, high levels of PD-L1 were identified in tumors arising from mesenchymal carcinoma human cell lines, whereas low levels of PD-L1 were evident in tumors generated by epithelial cell lines [30]. However, despite accumulating evidence supporting a link between PD-L1 expression and EMT, there is only a limited number of reports investigating PD-L1 and mesenchymal markers on patients’ CTCs [31,32]. 

Eribulin is a microtubule targeting anti-mitotic drug affecting microtubule dynamics by mechanisms distinct from most other tubulin targeting agents [33,34,35,36]. Eribulin has shown antitumor activity in many human xenograft models of various solid tumor types ([37] and references therein). In addition to its antimitotic action, eribulin has been suggested to operate via non-mitotic mechanisms on vascular remodeling, reversal of EMT to MET (mesenchymal to epithelial transition) [38,39], increasing differentiation, decreasing the capacity of cancer cells for migration and metastasis and decreasing the immunosuppressive environment ([40] and references therein). A limited number of studies have investigated the effect of eribulin on cancer tissue samples [39,41] or the number and mesenchymal phenotype of CTCs [42,43] in MBC.

In the current study, we developed a quadruple immunostaining procedure to quantify epithelial/mesenchymal markers and PD-L1 expression on single CTCs, and we applied this methodology to analyze CTCs obtained from MBC patients treated with eribulin. Our goal was to evaluate in parallel the epithelial/mesenchymal and PD-L1 status in single and clustered CTCs before and eight days after eribulin administration, in order to investigate the effect of eribulin on the number and phenotype of CTCs and to evaluate any potential correlations with patient outcome. 

## 2. Results

### 2.1. Expression Levels of Keratins, Vimentin and PD-L1 in Breast Cancer Cell Lines

**Figure 1:**
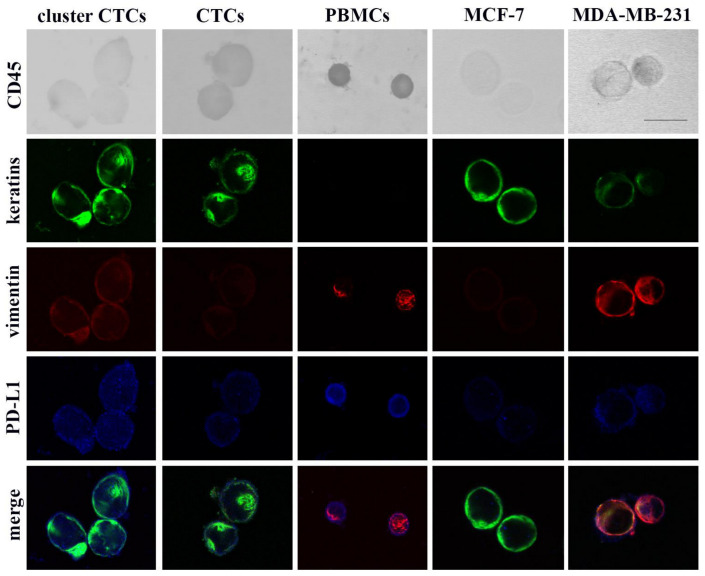
Quadruple immunostaining of PBMCs, CTCs and breast cancer cells. Representative images from cytospin preparations of PBMCs, CTCs, MCF-7 and MDA-MB-231 cells simultaneously stained for CD45, keratins, vimentin and PD-L1 are presented. Scale bar, 15 μm.

Representative images presented in Figure 1 show that breast cancer cells (CTCs, MCF-7 and MDA-MB-231 cells) do not stain for CD45 and express differential levels of keratins, vimentin and PD-L1. Specifically, MCF-7 cells show high keratins, low vimentin and low PD-L1 expression levels, while MDA-MB-231 cells are PD-L1 positive and show high vimentin and moderate expression of keratins. PBMCs from healthy volunteers expressed CD45, vimentin and PD-L1 and were negative for keratins. Therefore, our assay specified an “epithelial” cancer cell presenting the phenotype CD45^−^/keratins^+^/vimentin^low/−^ and a “mesenchymal” cancer cell expressing the CD45^−^/keratins^+^/vimentin^+^ phenotype. The epithelial or mesenchymal status of cells was further evaluated by measuring the vimentin/keratins (vim/ker) ratio among MCF-7 and MDA-MB-231 cells, respectively (see material and methods). Also, PD-L1 expression levels were measured in MCF-7 and MDA-MB-231 cells and the range of these values was used for subsequent CTC characterization (see material and methods).

### 2.2. Patient and Disease Characteristics

CTC analysis was performed before the initiation of treatment with eribulin (day 1) in 38 patients, as well as on day 8 of the first treatment cycle in 17 out of 38 patients, based on sample availability. Patient and disease characteristics are summarized in Table 1. Response to treatment and survival analysis was non-evaluable in 6 and 4 patients, respectively (Figure S1). Among the 34 patients who were eligible for survival analysis, 33 had progressed (median PFS: 2.3 months, 95%CI: 2.0–2.7) and 26 had died (median OS: 12.8 months, 95%CI: 4.8–20.7) at the time of analysis.

### 2.3. Detection and Characterization of CTCs Acording to EMT Status and PD-L1 Expression at Baseline 

**Figure 2:**
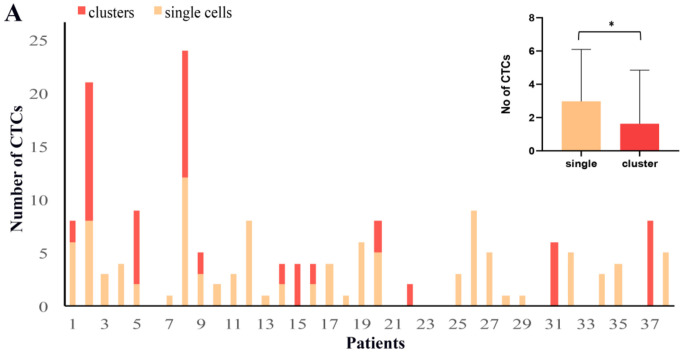

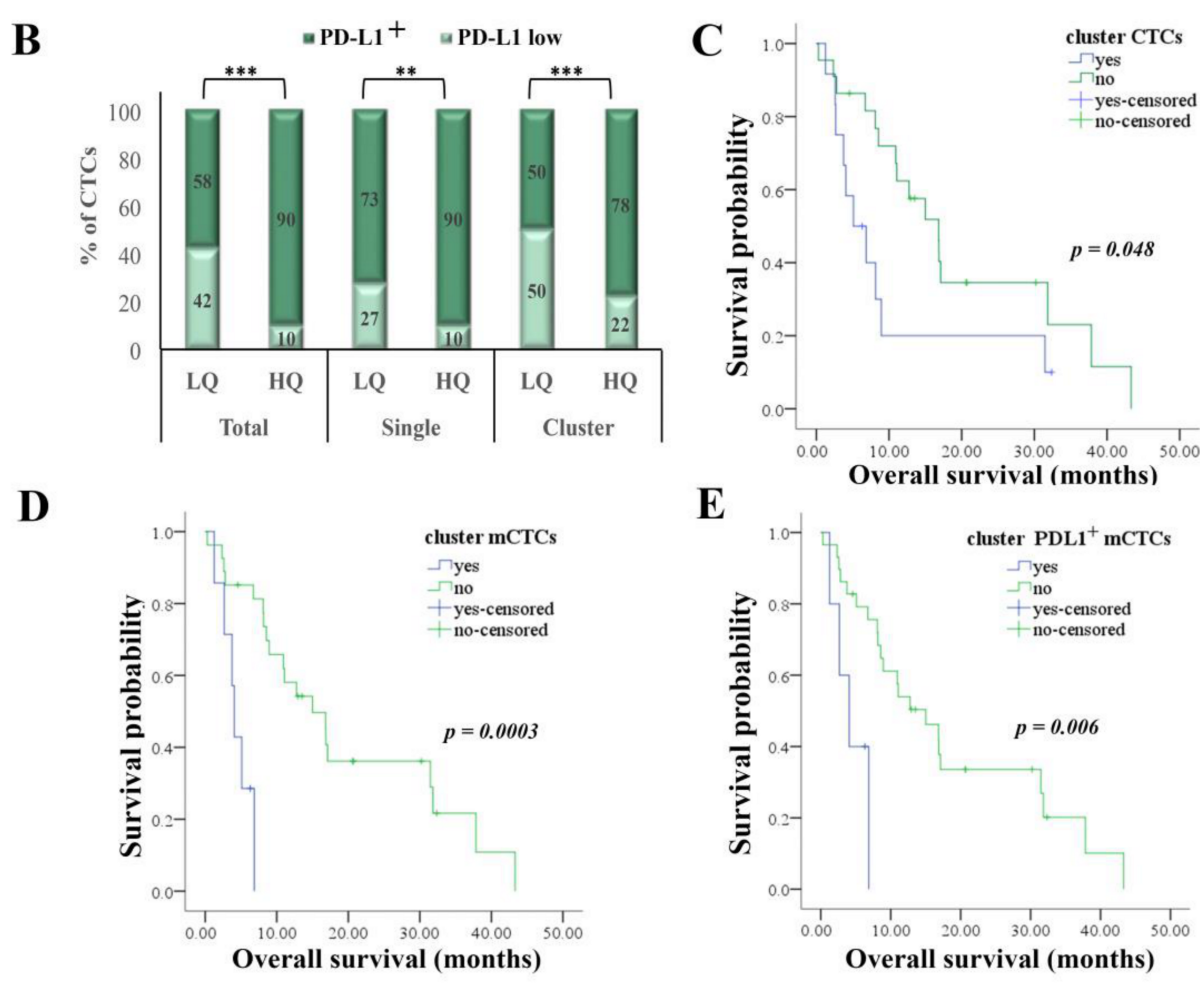
Detection and clinical significance of single and cluster subpopulations of CTCs. (**A**) Distribution of single and cluster CTCs in patients (*n* = 38). The number of CTCs per patient detected in all metastatic breast cancer patients is presented. Inset shows mean values of CTCs detected either as single cells or in clusters. * wilcoxon test; statistical significance at *p* = 0.037. (**B**) Frequency of PD-L1 expression (PD-L1^+^) in total, single and cluster CTCs within the low quartile (LQ) and the high quartile (HQ) of vim/ker ratios. Chi square test; statistical significance ** *p =* 0.032, *** *p* ≤ 0.001. (**C**–**E**) Kaplan-Meier analysis of overall survival (OS) according to the presence or not of cluster CTCs (**C**), cluster mCTCs (**D**) and cluster PD-L1^+^ mCTCs (**E**).

CTCs (DAPI positive, keratins positive and CD45 negative cells) were detected in 31 out of 38 (82%) patients (total number of CTCs: *n* = 173, median number of CTCs per patient: *n* = 4; range: 1–24). Representative images of the distinct CTC populations are depicted in Figure 1 and Figure S2. Single cells were identified in 71% of patients and represented 64% of total CTCs. Moreover, 17 CTC clusters consisting of two or more cells (range: 2–7 CTCs, see Figure S3) were found in 32% of patients and represented 36% of total CTCs. More CTCs were detected as single cells than in clusters (Wilcoxon mean no: 2.92 vs. 1.63, *p* = 0.037) (Figure 2A, inset). The intra-patient distribution of CTCs as single cells or in clusters is shown in Figure 2A.

The relative expression of keratins and vimentin varied among CTCs. According to the cut-offs of vim/ker ratios measured for MCF-7 and MDA-MB-231 cells, 54.9% of total CTCs were classified as epithelial CTCs (eCTCs) and 45.1% as mCTCs. Interestingly, the mesenchymal phenotype was enriched in clusters compared to single CTCs (in 59.7% and 36.9%, respectively, Chi square *p* = 0.0018).

Regarding PD-L1 expression, all but one patient had PD-L1 positive (PD-L1^+^) CTCs. At the CTC level, the majority of cells (80%) were PD-L1^+^, however PD-L1 expression numerically prevailed among mCTCs compared to eCTCs (in 80% and 67% of cells, respectively). Of note also, within the mCTCs population, PD-L1 expression prevailed among the high quartile (HQ) mCTCs compared to low quartile (LQ) mCTCs, regardless of their detection as total, single or cluster CTCs (Figure 2B).

### 2.4. Detection and Characterization of CTCs Acording to EMT Status and PD-L1 Expression on Day 8 of Eribulin Administration

To investigate the early effect of eribulin on the presence and phenotype of CTCs, blood samples from 17 patients were further analyzed on day 8 of the first-treatment cycle. CTCs were identified in 16 out of 17 (94%) patients before and in 12 out of 17 (71%) patients after eribulin treatment. A similar number (*n* = 106) of CTCs, as well as a similar number of patients harboring clustered CTCs (8 patients before and 9 patients after eribulin, see Figure S3) were identified in paired pre- and post-treatment samples. However, CTC counts varied before and after treatment in the majority of patients. Specifically, 6 (35%) patients showed increased, 10 (59%) decreased or undetectable (5 and 5 patients, respectively) and 1 (6%) patient unchanged number of CTCs. Even though eribulin did not affect total CTC counts (Wilcoxon mean no: 6.24 vs. 6.24, *p* = 0.856), a reduction in single CTCs and a significant increase in cluster CTCs (Chi square *p* = 0.009) was observed post-treatment (Figure S4). 

**Figure 3:**
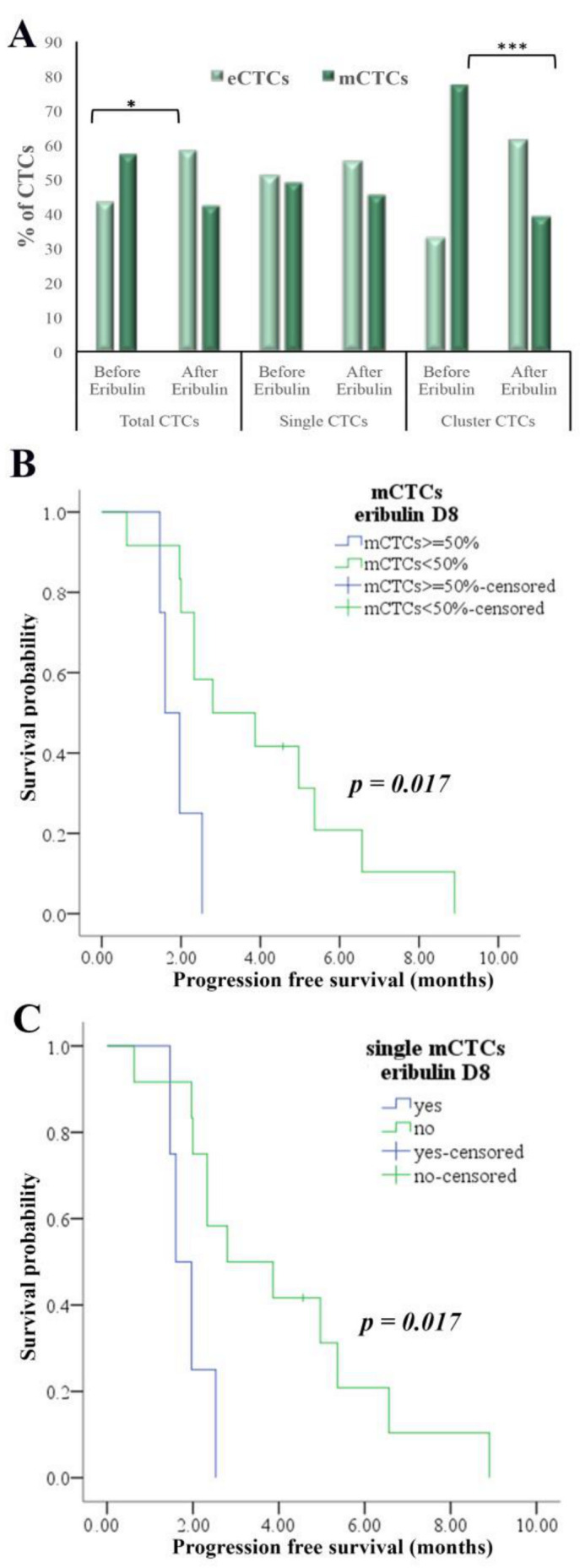
Detection and clinical significance of eCTCs/mCTCs subpopulations on D8 of the first eribulin cycle. (**A**) Percentages of eCTCs and mCTCs in total, single and cluster CTCs on days 1 (before eribulin) and 8 after the first administration of eribulin. Decrease in % of mCTCs in total CTCs (*p* = 0.047) and in cluster CTCs (*p* = 0.0001), are shown. Chi square test; statistical significance at the * *p* ≤ 0.05 and *** *p* ≤ 0.001. (**B**,**C**) Kaplan-Meier analysis of progression-free survival (PFS) according to the frequency of mCTCs (≥ 50% of total CTCs or not) (**B**) and the detection or not of single mCTCs (**C**).

Concerning the phenotype of CTCs, a reduction in mCTCs was observed in 10 out of 17 (59%) patients (Wilcoxon mean no: 7.39 vs. 6.13, *p* = 0.059). Specifically, eribulin significantly reduced the percentage of mCTCs in clusters (Chi square *p* = 0.0001) but not the single mCTCs (Figure 3A). This difference resulted from the significant increase in the number of eCTCs in clusters but not in single CTCs (Figure S5). At the patient level, among 8 patients with cluster mCTCs, 5 showed decreased and 3 increased cluster mCTCs (Wilcoxon mean no: 1.71 vs. 1.41, *p* = 0.58). 

**Figure 4:**
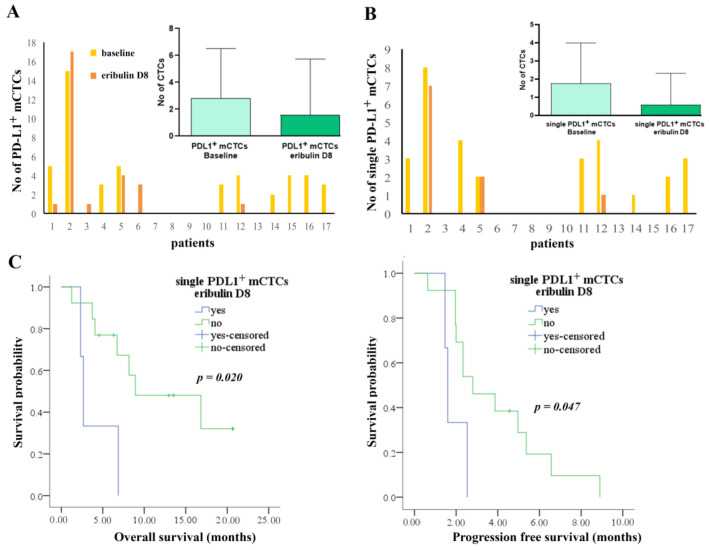
Detection and clinical significance of PD-L1^+^ mCTCs subpopulations on D8 of eribulin treatment. (**A**) Number of PD-L1^+^ mCTCs at baseline and on day 8 of the first administration of eribulin. Inset shows mean values of CTCs detected in patients (*p* = 0.034). (**B**) Number of single PD-L1^+^ mCTCs at baseline and on day 8 after the first administration of eribulin. Inset shows mean values of CTCs detected in patients (*p* = 0.008). (**C**) Kaplan-Meier analysis of overall survival (OS) and progression-free survival (PFS) according to the detection or not of single PD-L1^+^ mCTCs.

Eribulin had no effect in the number of PD-L1^+^ CTCs, however it was shown to decrease the mesenchymal PD-L1^+^ population (Wilcoxon mean no: 2.82 vs. 1.59, *p* = 0.034), and especially the single PD-L1^+^ mCTC subset (Wilcoxon mean no: 1.76 vs. 0.59, *p* = 0.008) (Figure 4A,B). Specifically, in 9 out of 12 patients (75%) reduced PD-L1^+^ mCTCs were detected post-treatment (Figure 4A), while in 8 out of 9 patients (89%) lower numbers of single PD-L1^+^ mCTCs (Figure 4B).

### 2.5. Correlation of CTC Monitoring with Clinicopathological Characteristics and Patient Outcome

The detection of cluster mCTCs at baseline was associated with hormone receptor-negativity (Chi square *p* = 0.022).

No association was observed between the detection or phenotype of CTCs and response to treatment at first evaluation. However, a different distribution was shown for cluster CTC subsets among patients experiencing progressive disease (PD) and those with partial response (PR) or stable disease (SD); cluster mCTCs and cluster PD-L1^+^ mCTCs were identified exclusively in PD patients (in 30% and 25% of patients, respectively), but in none of SD/PR patients. 

Reduced overall survival (OS) rates were confirmed among patients harboring cluster CTCs (median: 5.1 months vs. 18.6 months, *p* = 0.048) and especially cluster mCTCs (median: 4.1 months vs. 15 months, *p* = 0.0003) (Figure 2C,D). Combined analysis of PD-L1 and EMT status revealed a shorter OS among patients harboring cluster PD-L1^+^ mCTCs (median: 4 months vs. 15 months, *p* = 0.006) (Figure 2E). In a subgroup analysis based on the molecular BC subtype, the detection of cluster mCTCs or cluster PD-L1^+^ mCTCs was associated with reduced OS among hormone-receptor-positive patients only (*n* = 18; *p* = 0.004 for both CTC subsets), but not in the triple-negative patient cohort (*n* = 11; *p* = 0.268 and *p* = 0.634, respectively), whereas HER2-positive patients (*n* = 5) had not detectable any clusters of these specific phenotypes.

In Univariate Cox-regression analysis, the presence of central nervous system (CNS) metastases [(Hazard ratio (HR): 5.099, 95%CI: 1.476–17.616; *p* = 0.010)] and the baseline detection of cluster mCTCs (HR: 7.273, 95%CI: 2.103–25.147; *p* = 0.002) or cluster PD-L1^+^ mCTCs (HR: 4.964, 95%CI: 1.418–17.376; *p* = 0.012) were associated with increased risk of death (Table 2). However, the baseline detection of cluster mCTCs (HR: 5.145, 95%CI: 1.240–21.350; *p* = 0.024) was an independent marker associated with increased risk of death in Multivariate Cox-regression analysis (Table 3).

CTC evaluation on day 8 revealed an increase in single CTCs in a subset of PD patients only (30% of patients) but not in the SD/PR group. Moreover, single mCTCs and single PD-L1^+^ mCTCs were detectable at D8 exclusively in the PD patient group (in 30% and 20% of patients, respectively). Cluster eCTCs were more frequently evident in the SD/PR group (80%) compared to PD patients (10%).

Finally, CTC evaluation at day 8 revealed a lower PFS for patients harboring either single mCTCs (median: 1.6 months vs. 2.8 months, *p* = 0.017) or predominantly mCTCs (median: 4 months vs. 15 months, *p* = 0.017) (Figure 3B,C). Also, the presence of single PD-L1^+^ mCTCs was correlated with both reduced PFS (median: 1.6 months vs. 2.8 months, *p* = 0.047) and OS (median: 2.6 months vs. 8.9 months, *p* = 0.020) (Figure 4C). 

## 3. Discussion

In the current study, we evaluated the incidence of different CTC subpopulations characterized according to the EMT status and PD-L1 expression at baseline and on day 8 of the first administration of eribulin in patients with MBC. The results presented herein demonstrate that PD-L1 along with mesenchymal markers are co-expressed on CTCs, show that eribulin has an early effect on CTC status and suggest that the detection of specific PD-L1/EMT CTC subpopulations have prognostic implications in patients treated with eribulin.

PD-L1 expression has been associated with the EMT status of normal and malignant mammary cells and especially in claudin-low breast cancer cells [44]. Although the expression of mesenchymal markers or PD-L1 has been previously demonstrated in CTCs from patients with breast cancer [4,12,23,24,45], the co-expression of these markers at the single CTC level has not been reported so far. Herein we established a combined immunocytochemistry and immunofluorescence assay, which allowed the parallel evaluation of PD-L1 and epithelial and mesenchymal markers on individual CTCs. Using this novel approach, we showed that PD-L1 is expressed in both epithelial and mesenchymal CTCs, however it prevailed in the highly mesenchymal compared to the low mesenchymal CTC subpopulation. In accordance to our findings, in a previously published report, PD-L1 was highly expressed in epithelial (EpCAM positive) CTCs regardless of the type of cancer [45] and was co-expressed with mesenchymal markers in NSCLC CTCs [31,32]. 

Our study revealed high CTC-positivity (82%) of MBC patients with a variable range (1 to 24) of detected CTCs per patient (median number of CTCs per patient *n* = 4), indicating an effective CTC isolation procedure. Also, overestimation of keratins positive cells was avoided, since CD45 was used as an exclusion marker for PBMCs. We individually analyzed single CTCs and clusters, since accumulating data support distinct biological features and clinical relevance between these two CTC populations [4,6,7,46]. CTC clusters were detected in almost 40% of CTC-positive patients, and in accordance with previous reports in MBC and NSCLC, we observed that the EMT phenotype was more frequent among cluster than in among single CTCs [4,6,8].

Our results showed that the presence of cluster CTCs at baseline was associated with poor patient outcome, corroborating the previously reported prognostic value of cluster CTCs identified by using different methodologies in multiple types of cancer [5,6,7,8]. Importantly, we further showed that the detection of cluster mCTCs or cluster PD-L1^+^ mCTCs independently predicted for increased risk of death. This observation indicates that these characteristics may confer enhanced aggressiveness in cluster CTCs, underlining the necessity of future studies to phenotype the clusters. The combined evaluation of EMT status and PD-L1 on primary tumor tissue has been proposed as a prognostic biomarker in many types of cancer, such as breast, lung and colon cancer [28]. In accordance with our study, the co-expression of PD-L1 and EMT on CTCs was a predictor of shorter survival in patients with NSCLC [31]. 

Based on *in vitro* data concerning the effect of eribulin on MET transition in breast cancer cells [38], we examined the presence and phenotype of CTCs on day 8 following the first eribulin administration. Interestingly, the post-treatment phenotype of CTCs differed as compared to the baseline one. In particular, cluster mCTCs, but not single mCTCs, significantly decreased following eribulin treatment. These findings are in accordance with the previously reported preclinical observations on the eribulin-induced effects in mesenchymal breast cancer cells [38] and further suggest that eribulin may differentially affect the characteristics of CTCs according to their detection as single or cluster cells. Recently, binding sites for stemness- and proliferation-associated transcription factors have been found hypomethylated in CTC clusters from breast cancer patients, whereas dissociation of CTC clustering into single cells modified the DNA methylation state [46]. Cluster CTCs were differentially enriched in cell-cell adhesion, such as desmosome and adherent junction, markers [46,47] and the proliferation marker Ki67 [46]. Eribulin has a multidimensional mechanism of action, affecting among others cytoskeleton organization, cell proliferation and EMT [40], which is probably dependent on the cell type and the physiological and organizational state of the cells. Thus, given the molecular heterogeneity between cluster and single CTCs [7,46], a differential effect of eribulin, which could result in the acquisition of specific cell characteristics, is rather anticipated. These results highlight the importance for further phenotyping of single and cluster CTCs during treatment for their potential in the discovery of novel pharmacodynamic and/or predictive markers of treatment response and patient outcome.

We herein show that cluster mCTCs and cluster PD-L1^+^ mCTCs were detectable at baseline exclusively in patients experiencing PD at first evaluation of response to treatment. Moreover, single mCTCs and single PD-L1^+^ mCTCs were detectable on D8 exclusively in patients experiencing PD. Indications for the predictive significance of alterations in EMT status of CTCs during treatment with eribulin have been previously reported in metastatic breast cancer [42]. Among 6 patients with exclusively mCTCs at baseline, all three patients experiencing PR or SD had a decrease in mCTC detection rates as compared to none of the 3 patients who had PD. Moreover, the PD group tended to have more mCTCs. However in the same report, the timing of the second draw was not explicitly defined [42].

In the current study, a significant decrease in the number of PD-L1^+^ mCTCs was observed on D8, suggesting that eribulin may affect the expression of immune targets on CTCs. Similar results on the down-regulation of PD-L1 expression by eribulin were recently shown in tissue samples from metastatic breast cancer patients [41]. Nonetheless, the detection of either single mCTCs or PD-L1^+^ mCTCs after eribulin treatment was associated with shorter PFS. This finding implies that these CTC subsets may represent a resistant CTC population with increased potential in evading immune surveillance and driving disease progression [48]. However, further studies in larger patient cohorts are required to evaluate the association of the reversal in CTC phenotypes with patient outcome.

Strengths of our study include the establishment of a novel assay, which allowed the evaluation of the EMT status and PD-L1 co-expression in individual cells both before and early after eribulin administration. Using confocal microscopy, we defined the vimentin/keratins ratio at the single cell level in representative breast cancer cell lines and patients’ CTCs. Images of cluster CTCs were obtained at different confocal sections in order to distinguish individual cells within the cluster, thus allowing the evaluation of the different markers at the single cell level. Given the limited information available concerning the phenotype of cluster CTCs in breast cancer [4,46], the detection of distinct subpopulations associated with patient prognosis represents an important contribution. It should be noted however that the nature of our analysis is exploratory and in addition sample availability on day 8 of eribulin administration was restricted to a rather limited number of patients. These limitations preclude firm conclusions to be drawn regarding the associations between the different CTC phenotypes and of their alterations with response to treatment and patient outcome. 

In conclusion our study shows for the first time that cluster mCTCs or cluster PD-L1^+^ mCTCs detected at baseline are highly associated with poor patient outcome in patients with metastatic breast cancer treated with eribulin. Furthermore, it depicts the early effects of eribulin on CTC phenotypes and indicates that single mCTCs or single PD-L1^+^ mCTCs detected as early as 8 days post-treatment may have prognostic implications. These findings support the role of CTC analysis in the prediction of outcome and the monitoring of disease evolution in real time. However, it should be stressed that the clinical value of CTC detection and characterization as a prognostic tool for patients treated with eribulin merits further investigation in a larger cohort of patients. Since “MET” is likely to occur predominantly in cluster CTCs (data reported herein), proteins related to EMT/MET (stemness markers such as CD24, ALDH1) and those mainly expressed in cluster CTCs and possibly affected by eribulin treatment (cell proliferation such as Ki67 and cytoskeleton dependent cell adhesion such as E-Cadherin, desmoplakin, plakoglobin) also deserve further evaluation in future studies. Finally, analysis of CTC phenotypes at later time points, such as on the first evaluation of response to treatment and on disease progression, may uncover the long-term effects of eribulin and their role in predicting treatment outcomes and in revealing mechanisms of resistance to therapy.

## 4. Material and Methods

### 4.1. Study Design

The current prospective study included 38 patients with metastatic BC, who received eribulin as second or subsequent line of treatment at the Department of Medical Oncology of the University General Hospital of Heraklion (Crete, Greece) from 2016–2020. Eribulin was administered at the dose of 1.23 mg/m^2^ on days 1 and 8 of a 21-day cycle. Peripheral blood samples were obtained before the administration of eribulin on day 1 of the first treatment cycle; in 17 patients blood was also obtained on day 8 before drug administration. CTC detection and characterization was performed on peripheral blood mononuclear cells (PBMCs) cytospin preparations. Clinical characteristics and follow-up information for each patient were prospectively collected. This study was approved by the Ethics and Scientific Committees of the University General Hospital of Heraklion (2899/08-06-2016), Crete, Greece. All patients gave their written informed consent to participate in this study.

### 4.2. Cell Lines and Treatments

#### 4.2.1. Culture Conditions

MCF-7 (mammary adenocarcinoma) and MDA-MB-231 (human breast carcinoma) cell lines were obtained from American Type Tissue Culture Collection (Manassas, VA, USA). MCF-7 cells were cultured in Dulbecco’s modified Eagle’s medium (DMEM) plus 0.2 U/mL insulin and MDA-MB-231 in DMEM medium at 37 °C in a humidified atmosphere containing 5% CO_2_. Culture media were purchased from Biosera (Nuaille, France) and were supplemented with 10% heat inactivated fetal bovine serum, penicillin and streptomycin. 

#### 4.2.2. Cytospin Preparations of Cultured Cells

Cells were harvested by trypsinization, washed with PBS and aliquots of 500,000 cells were centrifuged at 2000 rpm for 2 min on glass slides. Cytospins were dried and stored at −80 °C before use. 

### 4.3. Confocal Microscopy

#### 4.3.1. Patients and Cytospin Preparations

Peripheral blood (10 mL in EDTA) was obtained from a group of 38 metastatic breast cancer patients on progression before the initiation of a new line of treatment and from 17 patients after 8 days of eribulin administration. Blood was collected by vein puncture after disposal of the first 5 mL in order to avoid contamination with epithelial cells from the patient skin. PBMCs were isolated after Ficoll-Hypaque (Sigma-Aldrich, St Louis, MO, USA) density gradient (d = 1.077 g/mL) centrifugation at 1800 rpm for 30 min, washed three times with PBS and centrifuged at 1500 rpm for 10 min. Aliquots of 1,000,000 cells were centrifuged at 2000 rpm for 2 min on glass slides. Cytospins were dried and stored at −80 °C for further use.

#### 4.3.2. Quadruple Immunostaining

We developed a method combining immunocytochemistry, indirect and direct immunofluorescence. Cytospins were fixed with 4% formaldehyde in phosphate buffered saline (PBS) for 5 min at room temperature and permeabilized with Triton X-100. Fixed cells were incubated in blocking buffer (PBS, pH 7.4, 0.2% Triton X-100 and 1% fish skin gelatin) and stained first for CD45 (mouse monoclonal, clone 2B11+PD7/26, Dako, Santa Clara, CA, USA) by immunocytochemistry using the UltraVision™ Quanto Detection System HRP DAB kit (Thermo Fisher Scientific, Fremont, CA, USA). Then cells were stained indirectly with primary antibodies for vimentin (mouse monoclonal, sc-6260, Santa Cruz Biotechnology, Dallas, TX, USA) and PD-L1 (rabbit monoclonal, E1L3N, Cell Signaling Technology Inc., Danvers, MA, USA) and the corresponding anti-mouse and anti-rabbit secondary antibodies labeled with Alexa 633 (blue staining, Thermo Fisher/Invitrogen, Carlsbad, CA, USA) and CF555 (red staining, Biotium, San Francisco, CA, USA) dyes and then directly with Alexa 488-labelled primary antibodies (AE1/E2 53-9003-82, Invitrogen, Carlsbad, CA, USA and C11 NBP1-48348AF-488, Novus Biologicals, Centennial, CO, USA) for most keratins, overnight at 4 °C. The titration for optimal activities and the specificity of each antibody was evaluated using the MCF-7 and the MDA-MB-231 cell lines spiked into normal donor PBMCs. In each separate experiment, positive samples for all markers and negative controls prepared by omitting the respective primary antibody, to exclude non-specific binding, were included.

#### 4.3.3. Identification and Phenotypic Characterization of CTCs

All cytospin preparations of PBMCs were first examined under a conventional epifluorescence microscope (Leica) using 40× objective lens with oil immersion and were further analyzed by confocal (Leica SP) microscopy. To prevent any signal interference (green, red and blue) generated by the different emission spectra, the detection of each one of the markers was performed by sequential laser confocal scan. Fixed confocal settings were used for all specific measurements. Images were taken from all CTCs detected (DAPI positive, keratins positive and CD45 negative cells) and were stored electronically. As positive controls, cytospins of MCF-7 and MDA-MB-231 cells spiked into normal donor PBMCs were included in each separate experiment.

For the characterization of CTCs associated in clusters multiple confocal series of images were analyzed to visualize and quantify the expression of the different markers of CTCs in different optical fields [4].

To determine the epithelial or mesenchymal status of CTCs, we used the range of vim/ker values for MCF-7 and MDA-MB-231 cells. These ratios were calculated using the expression levels of keratins and vimentin measured in at least 100 cells. The range for MCF-7 (0.01 to 0.28) and for MDA-MB-231 (0.88 to 22.5) defined the epithelial and the mesenchymal cancer cells. CTCs exhibiting ratios up to 0.88 were characterized as eCTCs, whereas values greater than this defined as mCTCs. 

PD-L1 expression was determined on CTCs by using the differential expression values (CTCF, arbitrary units) measured in at least 100 MCF-7 (range: 10 to 40) and MDA-MB-231 (range: 60 to 350) cells as cut-offs. CTCs showing values up to those measured for MCF-7 cells were characterized as negative or low PD-L1 expressing cells, whereas those exhibiting values higher than these and within the range in the population of MDA-MB-231 cells defined as PD-L1 expressing cells.

### 4.4. Image Analysis

To quantify the fluorescence intensity of the markers of interest, images were subjected to java-based image processing with the use of ImageJ program (NIH). All CTCs identified on patient cytospins and representative images (100 confocal specimens) of each MCF-7 and MDA-MB-231 cells were analyzed using ImageJ. Fluorescence intensity was expressed as Corrected Total Cell Fluorescence (CTCF). 

### 4.5. Statistical Analyses

Chi square test was used to compare total CTCs or CTC subtypes and to correlate with clinical data. Wilcoxon test was used to compare CTCs/patient and their subtype variables. Kaplan-Meier survival analysis was used to estimate the probability of relapse and death over time. PFS was calculated from the date of the treatment initiation to the date of disease progression or death from any cause; OS was calculated from the date of the treatment initiation to death from any cause. The log-rank test was used to compare survival curves between groups. Univariate Cox regression analysis along with a multivariate Cox proportional hazards regression model, were performed to investigate the associations between different parameters and the risk for relapse and death. All tests were two-sided, and *p* values were considered significant at the 0.05 level. All analyses were performed using the SPSS20 program. 

## 5. Conclusions

Our data support the importance of CTCs expressing mesenchymal or co-expressing mesenchymal and PD-L1 characteristics for the outcome of patients treated with eribulin. Also, although in a limited number of patients, our study highlighted the necessity of phenotyping CTCs early after eribulin administration. Eribulin most likely induced “MET” in cluster mCTCs and significantly decreased PD-L1 expression in single mCTCs. Most probably, eribulin exerts on CTCs other not yet identified actions, since specific cluster and single CTCs subpopulations exposed to eribulin showed a different behavior on patient outcome. Further phenotypic analysis of CTCs, including, among others, stem and proliferation markers, in a larger cohort of patients would help to identify more specific subpopulations important for patient outcome and shed light on the mode of action of eribulin therapy. 

## Figures and Tables

**Table 1:** Patient and disease characteristics.

Patients (*n* = 38)	*n* (%)
Age (years), median (range)	61 (34–78)
Performance Status (PS)	
0–1	30 (78.9)
2	8 (21.1)
Menopausal status	
pre-menopausal	13 (34.2)
post-menopausal	25 (65.8)
Histology	
Ductal	31 (81.6)
Lobular	3 (7.9)
Mixed	2 (5.3)
Other	2 (5.3)
Stage at diagnosis	
I–III	30 (78.9)
IV	8 (21.1)
Subtype	
ER+ and/or PR+/HER2-	20 (52.6)
Triple-negative	12 (31.6)
HER2+	6 (15.8)
Surgery *	
Yes	31 (81.6)
No	7 (18.4)
Prior chemotherapy for metastatic disease	
Taxane-based	16 (42.1)
Anthracycline-based	1 (2.6)
Taxane/Anthracycline -based	15 (39.5)
Other	3 (7.9)
None	3 (7.9)
Prior Trastuzumab for metastatic disease	
Yes	6 (15.8)
No	32 (84.2)
Prior Hormonotherapy for metastatic disease	
Yes	20 (52.6)
No	18 (47.4)
Disease sites	
1–2	20 (52.6)
>2	18 (47.4)
Organs affected	
Bones	24 (63.2)
Liver	23 (60.5)
Lung	18 (47.4)
Central Nervous System	6 (15.8)
Lymph nodes	14 (36.8)
Cutis	5 (13.2)
Line of Eribulin treatment	
1–2	13 (34.2)
>2	25 (65.8)
Response to treatment at first evaluation	
Partial response	3 (7.9)
Stable disease	9 (23.7)
Progressive disease	20 (52.6)
Non-evaluable	6 (15.8)
Disease status at the end of treatment	
Partial response	1 (2.6)
Stable disease	9 (23.7)
Progressive disease	22 (57.9)
Non-evaluable	6 (15.8)

Abbreviations: ER, estrogen receptor; PR, progesterone receptor. * Surgery refers to removal of the primary tumor and of axillary lymph nodes.

**Table 2:** Univariate Cox regression analysis for PFS and OS (Patients: *n* = 34).

Univariate Cox Regression Analysis	Progression-Free Survival (PFS)		Overall Survival (OS)	
Covariates	HR (95% CI)	*p* Value	HR (95% CI)	*p* Value
Age (>median 59 yrs)	1.420 (0.648–3.111)	0.381	1.052 (0.420–2.635)	0.914
Performance status (0–1)	0.503 (0.199–1.272)	0.147	0.403 (0.147–1.110)	0.079
Menopausal status (pre)	1.621 (0.770–3.410)	0.203	1.326 (0.592–2.970)	0.493
Stage at diagnosis (I–III)	1.506 (0.618–3.666)	0.367	1.593 (0.540–4.701)	0.399
Molecular subtype of tumor				
ER+ and/or PR+/HER2-	0.349 (0.156–0.778)	0.010 *	0.452 (0.200–1.024)	0.057
HER2-positive	0.735 (0.250–2.160)	0.575	0.448 (0.103–1.951)	0.285
Triple-negative	2.866 (1.285–6.393)	0.010 *	2.211 (0.976–5.007)	0.057
No of organs affected (>2)	1.188 (0.590–2.391)	0.629	1.787 (0.758–4.211)	0.184
Metastatic sites				
Liver	1.290 (0.634–2.625)	0.483	1.256 (0.534–2.953)	0.601
Lung	0.851 (0.424–1.710)	0.651	0.826 (0.374–1.823)	0.636
Bones	1.230 (0.574–2.637)	0.594	1.507 (0.664–3.421)	0.327
Lymph nodes	0.722 (0.336–1.549)	0.402	0.486 (0.199–1.190)	0.114
CNS	1.848 (0.624–5.475)	0.268	5.099 (1.476–17.616)	0.010 *
Skin	1.096 (0.378–3.178)	0.867	3.399 (0.919–12.569)	0.067
Line of eribulin treatment (>2)	1.139 (0.554–2.341)	0.724	0.804 (0.352–1.833)	0.603
Νew visceral metastases	2.670 (0.800–8.906)	0.110	1.390 (0.526–3.676)	0.507
cluster CTCs	1.119 (0.537–2.334)	0.764	2.266 (0.8986–5.211)	0.054
cluster mCTCs	1.728 (0.720–4.147)	0.221	7.273 (2.103–25.147)	0.002 *
cluster PD-L1^+^ mCTCs	1.876 (0.705–4.995)	0.208	4.964 (1.418–17.376)	0.012 *

The reference category is in the bracket. * Statistical significance at the *p* ≤ 0.05 level.

**Table 3:** Multivariate Cox regression analysis for OS (Patients: *n* = 34).

Multivariate Cox Regression Analysis	Overall Survival (OS)	
Covariates	HR (95% CI)	*p* Value
Central nervous system metastasis	2.121 (0.502–8.960)	0.306
Cluster mCTCs	5.145 (1.240–21.350)	0.024 *

* Statistical significance at the *p* ≤ 0.05 level.

## Supplementary Materials

The following are available online at https://www.mdpi.com/2072-6694/12/12/3735/s1, Figure S1: Flow chart showing analysis of patients treated with eribulin, Figure S2: Phenotypic analysis of cluster CTCs, Figure S3: Distribution of CTCs in clusters, Figure S4: Changes in single and cluster CTCs at D8 of eribulin treatment, Figure S5: Detection of eCTCs/mCTCs subpopulations on D8 of the first eribulin cycle.
