# Supplementary material for: Epithelial/Mesenchymal Characteristics and PD-L1 Co-Expression in CTCs of Metastatic Breast Cancer Patients Treated with Eribulin: Correlation with Clinical Outcome
Source: Cancers (Basel). 2020 Dec 11;12(12):3735. doi: 10.3390/cancers12123735 (PMC7764288; doi:10.3390/cancers12123735)
Supplement: Supplementary file 1 [file cancers-12-03735-s001.pdf]

cancers

Epithelial/Mesenchymal Characteristics and PD-L1 Co-Expression in CTCs of Metastatic Breast Cancer Patients Treated with Eribulin: Correlation with Clinical Outcome

Hara Polioudaki, Anastasia Mala, Eleni Gkimprixi, Maria A. Papadaki, Amanda Chantziou, Maria Tzardi, Dimitris Mavroudis, Sofia Agelaki and Panayiotis A. Theodoropoulos

Supplementary Materials

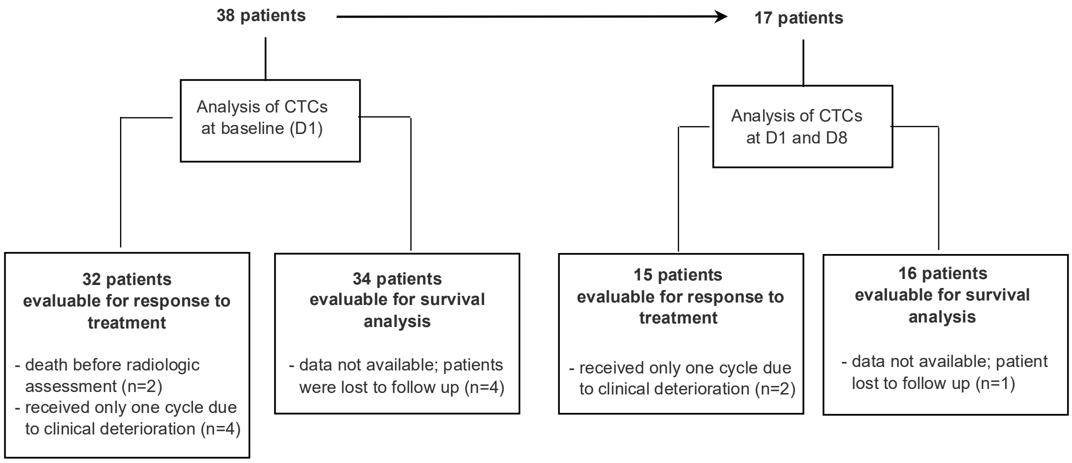

Figure 1. Flow chart showing analysis of patients treated with eribulin. CTCs were analyzed at baseline (D1) in 38 patients and both at baseline (D1) and after 8 days of first administration (D8) in 17 patients.

Cancers **2020**, *12*, 3735; doi:10.3390/cancers12123735 www.mdpi.com/journal/cancers

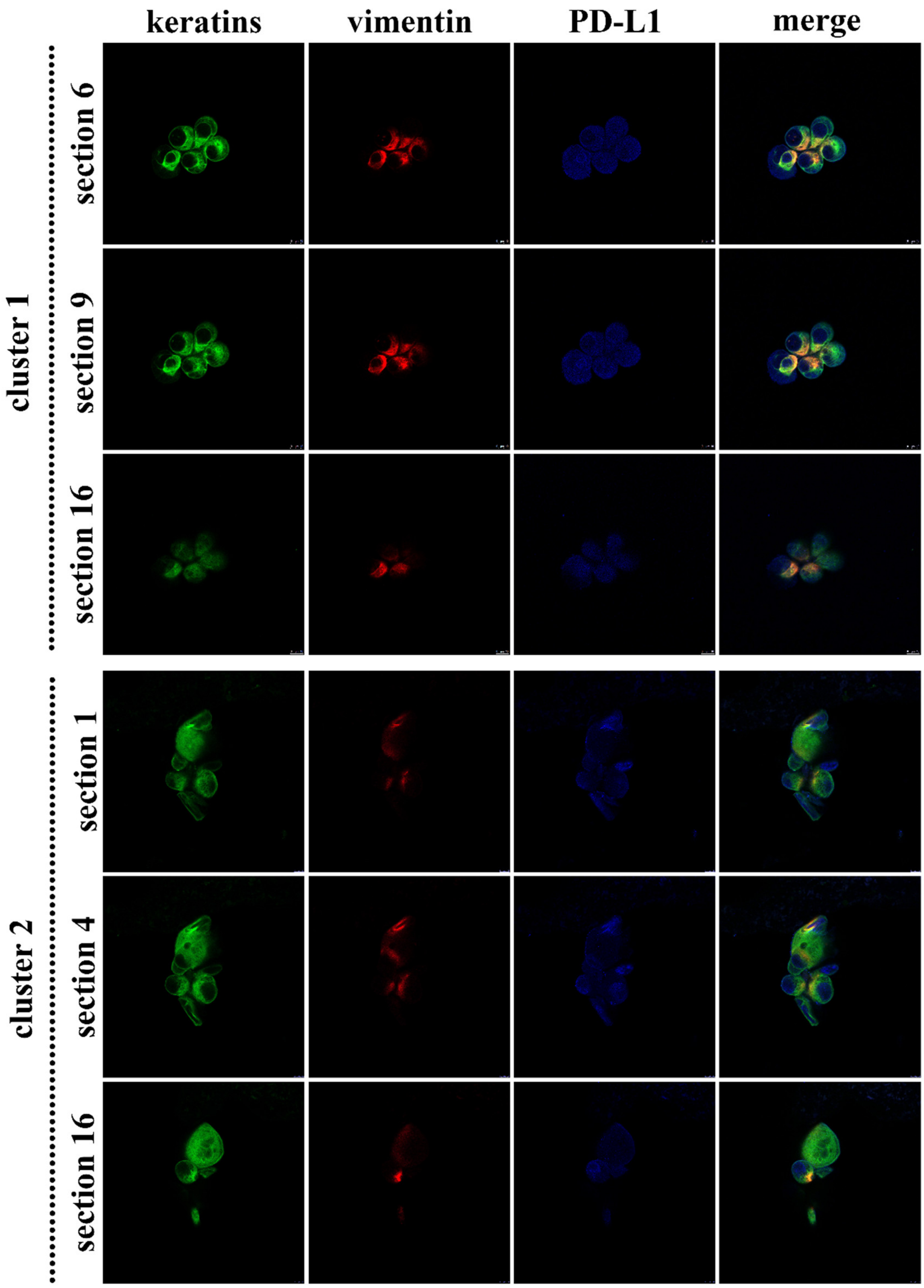

Figure 2. Phenotypic analysis of cluster CTCs. The immunostaining of the different CTCs in 2 clusters are shown in three different focal plans.

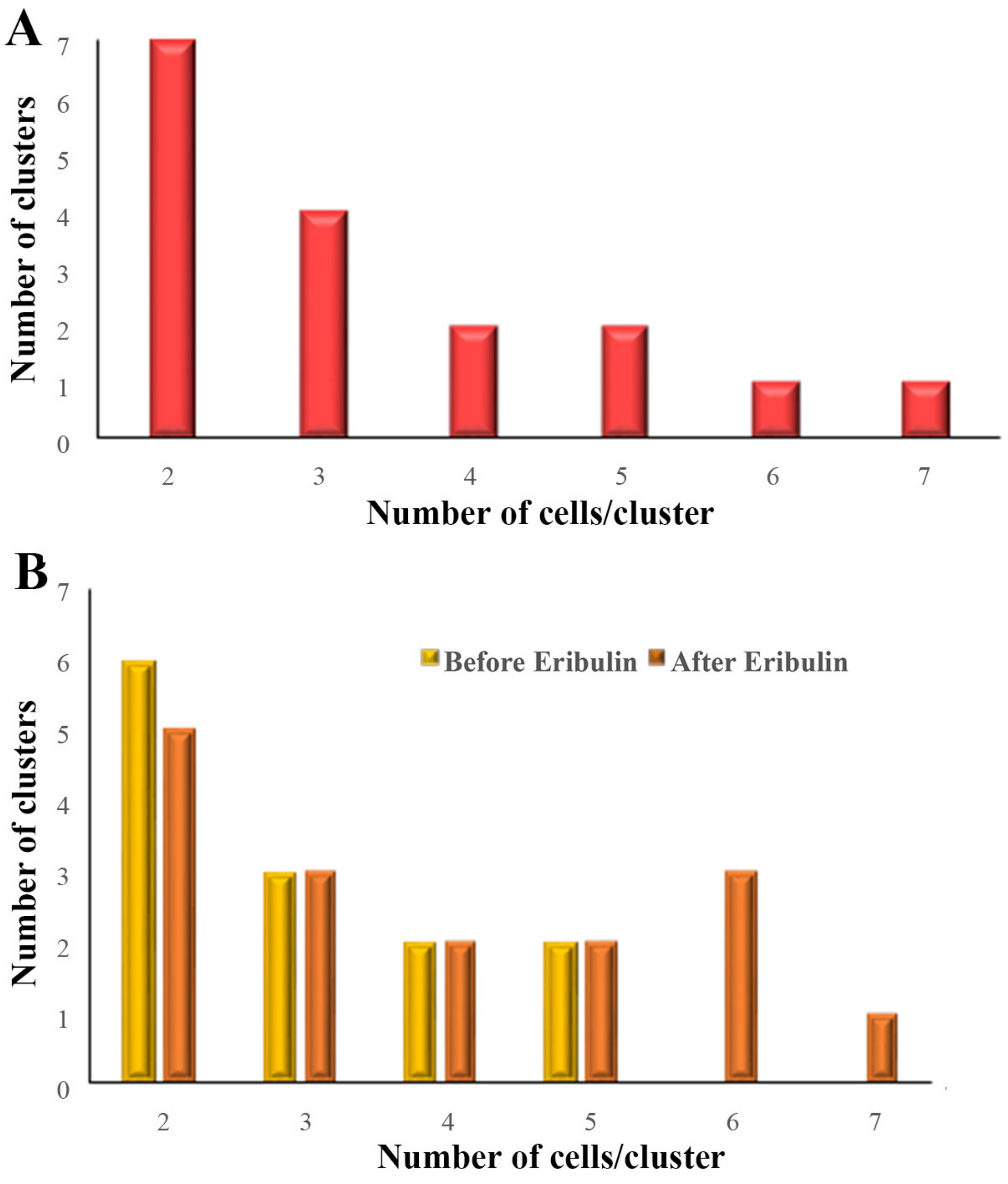

Figure 3. Distribution of CTCs in clusters. Frequency of CTCs in clusters at baseline (A) and before or after eribulin treatment (B).

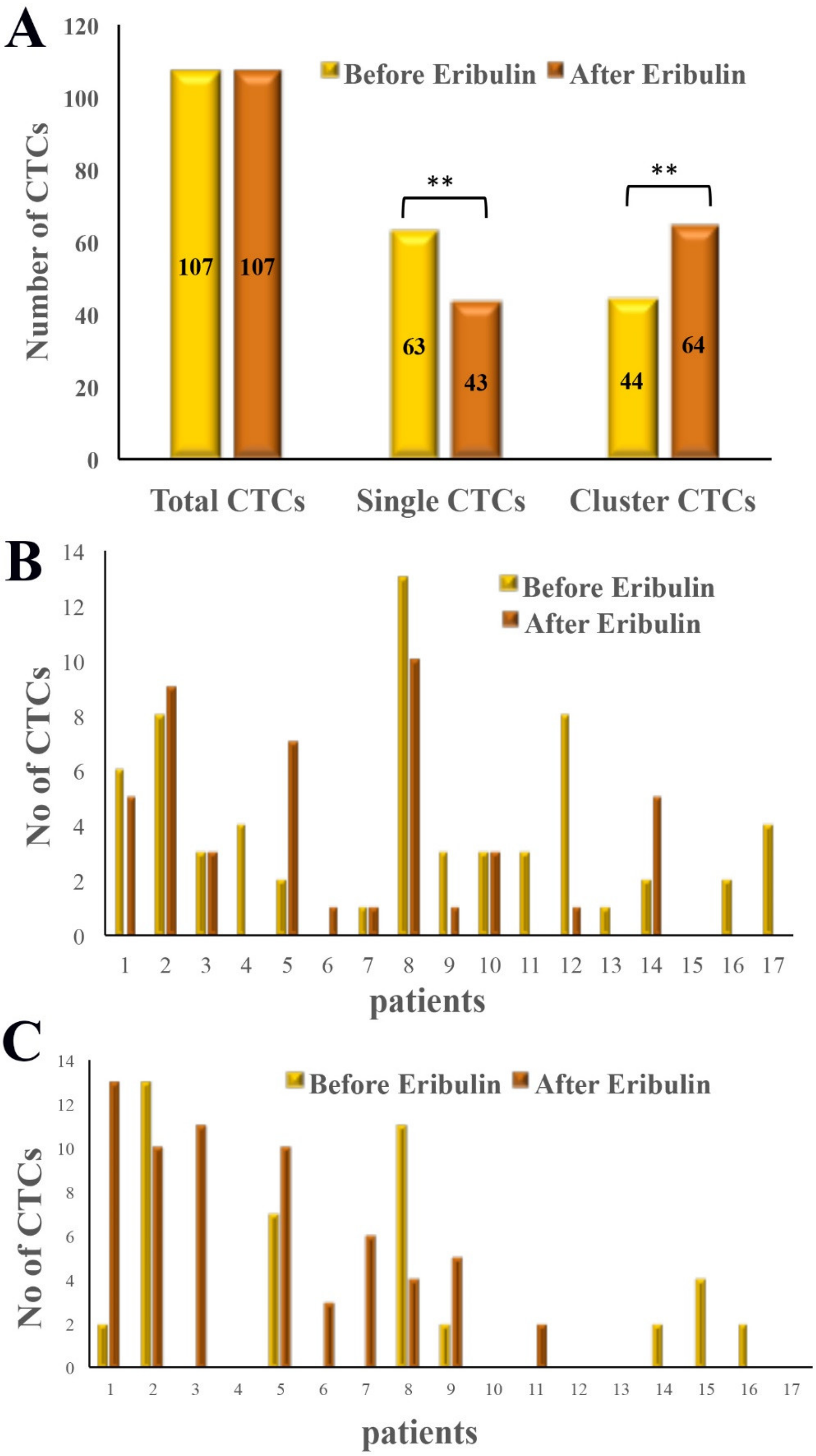

Figure 4. Changes in single and cluster CTCs at D8 of eribulin treatment. (A) Number of total, single and cluster CTCs at base line (before eribulin) and 8 days after the first administration of eribulin (after eribulin) ** Chi square test; statistical significance at the $p \leq 0.01$ level. Number of single (B) and cluster (C) CTCs detected in patients before and 8 days after the first administration of eribulin.

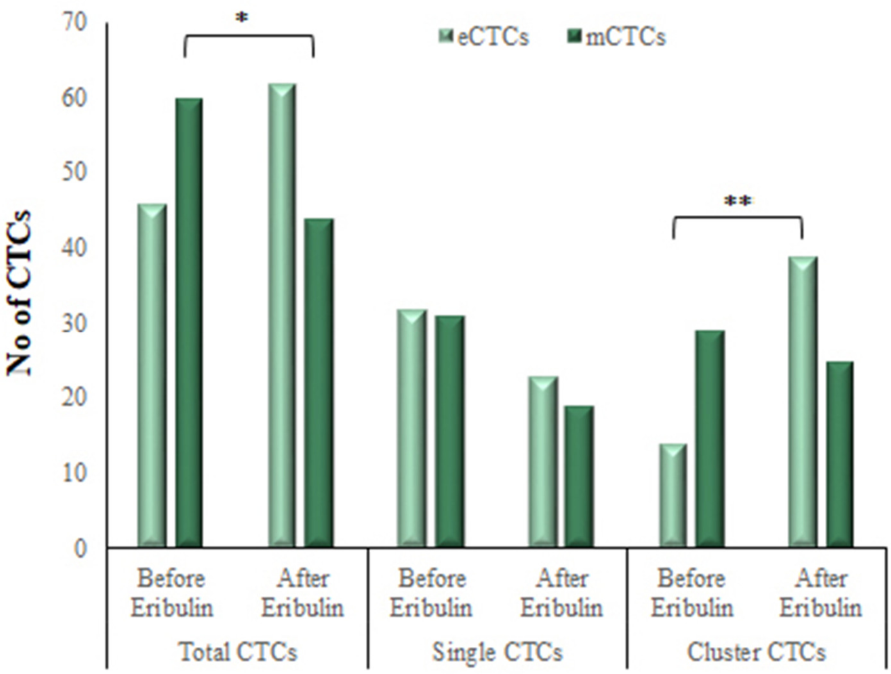

Figure 5. Detection of eCTCs/mCTCs subpopulations on D8 of the first eribulin cycle. Number of eCTCs and mCTCs in total, single and cluster CTCs on days 1 (before eribulin) and 8 after the first administration of eribulin. Decrease in the number of mCTCs in total CTCs ($p = 0.039$) and increase in the number of cluster eCTCs ($p = 0.0056$), are shown. Chi square test; statistical significance at the * $p \leq 0.05$ and ** $p \leq 0.01$.

Publisher's Note: MDPI stays neutral with regard to jurisdictional claims in published maps and institutional affiliations.

© 2020 by the author. Licensee MDPI, Basel, Switzerland. This article is an open access article distributed under the terms and conditions of the Creative Commons Attribution (CC BY) license (http://creativecommons.org/licenses/by/4.0/).
